# Annotations

**Published:** 1888-04-28

**Authors:** 


### ANNOTATIONS.

**A Homœopathic Earl.**

THE zeal of converts is not always tempered by discretion. Earl Dysart, who seems to be a homœopath, has made a very amusing offer to the managers of the Grantham Hospital—an offer which must have put the local medical men into a fine pickle. The noble earl, with beautiful simplicity, has offered £250 as a donation to the Enlargement Fund of the Hospital, and £100 a-year for ten years to the Maintenance Fund. A generous offer surely in these bad times; but mark the condition which accompanies it! It is that a homœopathic practitioner shall be appointed to the hospital staff, with full power to treat patients homœopathically. If the whole hospital can be converted to homœopathy his enthusiastic lordship will give £200 a-year. This is really a state of affairs which has more in it than appears on the surface. Here is a huge bribe offered to hospital managers by a man who wants them to agree with his decision on a subject about which they cannot scientifically know anything at all, and concerning which he must be quite as ignorant as they are. It may be presumed that Earl Dysart knows a little about fox-hunting. What would he think if a rich London tailor, who had never seen a pack of hounds in his life, were to offer two hundred a-year to the master of the local pack on condition that he should hunt foxes with ladies' poodles? Now, it will be admitted that the science and art of fox-hunting may be learnt as easily as the science and art of medicine: medical men and people generally will say "very much more easily." Does Earl Dysart really know anything more about scientific medicine than the London tailor knows about fox-hunting? The committee appear to have discussed the proposal with commendable gravity, and one member pointed out that in a New York hospital a third of the accommodation was apportioned to homœopathic patients. Nobody can stand against the changes of the times. If all the world determines that it knows more about medicine than medical men, and refuses to be treated except in the way it chooses for itself, physicians, except those who happen to be troubled with the vice of honesty, must of course give in, and consent to be guided by the superior wisdom of their patients. The honest ones, of which we may hope a few will be found, will consider it necessary to adopt some other calling. Lord Dysart has probably heard of the *reductio ad absurdum*. Has he sufficient logic to know when his own argument is put into that category? If doctors do not know better than earls and other members of hospital committees what science points out as the right lines of medical practice, they must be the stupidest of all God's intelligent creatures.

**Something like a Secretary.**

A SCOTCHMAN is nothing if not thorough. The inhabitants of Orkney, a remote island lying towards the land of the midnight sun, sends a large number of patients annually for treatment to the Edinburgh Royal Infirmary. They send the patients, but not the money for their maintenance. Mr. Trainer, the secretary to the infirmary, does not believe in this kind of thing. He has a great regard for fair play. Of Mr. Trainer's opinion is Dr. Clouston, the amiable and intelligent superintendent of the famous Morningside Asylum. Dr. Clouston himself is an Orkney man, and is evidently a little ashamed of his fellow-countrymen. But the Orcadians are no longer to be allowed to pick all the plums out of the Edinburgh Infirmary cake. Mr. Trainer has visited Orkney for the purpose of giving them that serious remonstrance and full instruction in moral duty which every Scotchman, being a born preacher, so well knows how to do. Dr. Clouston, too, has sent a brotherly epistle, in which the facts of the case are set forth with much plainness of speech. "I find," says the doctor, "that during the past five years 157 patients from Orkney were admitted. In that time, I regret to say, that only £69 was contributed from the county, or at the rate of 8*s*. 9*d*. per patient. . . . . Our 157 Orcadians were treated at a cost of £612, or £543 over the £69 contributed from the county. This money came out of the pockets of the benevolent elsewhere. . . . . We cheerfully support our churches, our poor, and our insane. Our sick should not be neglected." Mr. Trainer has undertaken a two days' voyage by sea to Orkney in order to stir up the Orcadians, and to organise a regular system of church collections, house-to-house contributions, and other methods of raising money likely to be acceptable to the inhabitants. This is surely the way to set about the business! The secretary will thus become known to every charitable contributor to the funds of the hospital, and will establish a personal connexion between donors and charity which may prove invaluable. People will give to *persons* whom they trust much more readily than to institutions, which to many are merely names. The personal element has a potency whose value it is impossible to exaggerate. We heartily wish for Mr. Trainer and his faithful henchman all the success which their ardent devotion to the interests of the hospital so well deserve. Other hospital secretaries might very well take a leaf out of this book.

**Hospital Patients and Stimulants.**

COMPLAINTS are often made that wines, spirits, and beer are ordered very freely for hospitals, and it is sometimes hinted that the patients do not get quite every bottle of wine and every glass of spirits for their own special use and benefit. We have no objection to such criticisms when they are just. On the contrary, we think they are too seldom offered; and on occasions very serious faults are allowed to pass unnoticed because kind-hearted committeemen hate to "make a row." Sometimes, however, a brave outsider comes to the rescue; and if he happens to be a man with a "bee in his bonnet," and knows very little about the subject, he is all the more likely to speak with confidence and authority. A case in point has just occurred at Nottingham. The annual meeting of the large and important hospital of that town was held in March, and since the publication of the Report a correspondence has taken place in the local press in which it has been affirmed that there is too much wine drunk at the hospital, that the patients don't drink it all, and that those who subscribe will do well to stop the supplies. The wise gentleman or lady who writes thus probably does not expect to be taken seriously. Even he might have the sense to know that the stopping of the supplies would entirely miss the object he aims at. It would punish the poor patients for the supposed fault of the officials. That is a kind of vicarious suffering which cannot be approved by any system of theology or law. But is it really the case that the expenditure of the hospital has been extravagant, and that wines have been used in excessive quantities? Emphatically not. We have had sent to us the annual Report, together with a detailed statement of accounts. According to this, the annual cost per occupied bed for the present year has been under £60, an amount which will compare favourably with the cost per bed of any similar hospital in the kingdom, and which is fully a third less than the cost per bed of some metropolitan hospitals. So much for general expenditure. Turning to the question of wines; the total amount expended during the year was £52 16*s*. 9*d*. The total number of in-patients treated was 1,413. So that, supposing the patients to have drunk every drop of wine which went into the building, each one of them would have had rather less than sixpennyworth. If that be either extravagant expenditure or a source of danger to the patient, we have nothing more to say. In addition to this there was a sum of £48 9*s*. spent on spirits, which, being divided among all the in-patients, would give about fivepennyworth to each. Or, putting the two items of stimulant together, each in-patient at the Nottingham Hospital has had considerably less than a shilling of charitable money spent upon him in this particular. And yet somebody, who is surely either joking or crazy, has boldly addressed the public and asked them to withdraw their subscriptions from an institution showing such careful management as this! We do not need to express an opinion; the figures both for the whole expenditure and for its separate details are more than satisfactory, and reflect the greatest credit alike upon the matron, the secretary, the dispenser, and all concerned.

